# Meals on Wheels and Family Caregiving: Benefits Received and Unmet Needs

**DOI:** 10.1093/geroni/igaf122.479

**Published:** 2025-12-31

**Authors:** Emily Gadbois, Nichole Stetten, Laura Samuel, Kimberly Bernard, Em Balkan, Yiqing Qian, Kali Thomas

**Affiliations:** Brown University School of Public Health, Providence, Rhode Island, United States; Brown University, Providence, Rhode Island, United States; Johns Hopkins University, Baltimore, Maryland, United States; Brown University, Providence, Rhode Island, United States; Brown University, Providence, Rhode Island, United States; Johns Hopkins University School of Nursing, Baltimore, Maryland, United States; Johns Hopkins University, Baltimore, Maryland, United States

## Abstract

Long-term services and supports are frequently provided by millions of unpaid family caregivers to community-dwelling older adults. Meals on Wheels programs provide home-delivered meals that help older adults continue to live in their communities. The benefits of Meals on Wheels programs to their clients are well-documented, but impacts on caregivers of those clients are less clear. In-depth, semi-structured interviews were conducted with 85 clients and 14 caregivers from 13 Meals on Wheels programs. Through these 99 interviews, we learned about the care and assistance provided by unpaid caregivers which included food-related support (acquiring and/or paying for groceries, cooking, initiating Meals on Wheels), physical care and assistance with activities of daily living and instrumental activities of daily living, transportation, medication and healthcare management, and financial support. Although clients and caregivers reported that Meals on Wheels eased some caregiving burden, they also identified substantial unmet needs. Many clients expressed that they would benefit from additional meals, as well as financial, transportation, and hands-on support. Caregivers reported substantial burden, and suggested that they would benefit from assistance with financial and care planning, identifying formal supports, and a desire for respite care. While Meals on Wheels provide essential nutrition support to older adults, this is just one component of need and its services are not intended to address all the care needs of older adults or their caregivers. This research is responsive to the Recognize, Assist, Include, Support, and Engage (RAISE) Family Caregivers Act and the associated National Strategy to Support Family Caregivers.

